# Asthma and Its Treatment

**Published:** 1910-03-05

**Authors:** J. E. Squire


### Hospital Clinics.

### ASTHMA AND ITS TREATMENT.

By J. E. SQUIRE, C.B., M.D., F.R.C.P.

(Delivered at the Polyclinic, Chenies Street, London, on Thursday, January 13, 1910.)

Gentlemen,—The term asthma is used rather laxly. Amongst the general public it is held to cover almost any case of urgent dyspnœa, so that we hear a person who has emphysema being spoken of as asthmatic. We, however, restrict the term more, though even medical practitioners use it rather widely. We employ the term in connection with urgent paroxysmal dyspnœa, and especially those cases which tend to frequent recurrence. But even so, the term covers a variety of different conditions. We speak, for example, of cardiac dyspnœa, the urgent dyspnœa which occurs so often in certain nerve states associated with heart lesions. That is not true asthma. So, too, in renal complaints, we sometimes find attacks of urgent dyspnœa which are spoken of as asthmatical—renal asthma—which, again, are not true asthmatic attacks.

### Incidence.

If we restrict the term, as I propose to do, chiefly to what is called true asthma, sometimes spasmodic asthma, we find there are certain cases which we might almost speak of as idiopathic asthma. There are others for which we use the term symptomatic asthma. In the first, we mean asthmatic attacks, the obvious cause of which is not easily determined. And in the second case we speak of asthmatic attacks occurring in the course of, or immediately following, some definite illness of which we are aware, and of which the symptoms are sufficiently evident. When we examine the causes of asthma, we have first of all to remember that, like some other nerve conditions, epilepsy, for example, it seems to be largely hereditary, or, let us say rather, that it occurs largely in individuals belonging to neurotic families. It may be that the parents, or one of them, was subject to epilepsy, and the second generation shows one or two cases of asthma. Undoubtedly this hereditary tendency shows in a large proportion of the cases.

We next come to the question of sex. Asthma is more common amongst men than in women; it is said in the proportion of two to one. The exact cause of that it is difficult to say, as men are considered to be far less neurotic than women. But there may be two sides to that question. At any rate, the neurosis of the woman does not generally manifest itself as asthma. True asthma may occur at almost any age. It is found in young children, it is not uncommon after five years of age, and it has been met with even in infants. In middle age it is common, and is not rare in old age. The predisposing conditions, in addition to heredity, are diseases which are weakening and leave behind them some want of stability of the nervous system; for instance, influenza, which does cause very marked disturbance of the nervous system, may lead to asthmatic attacks recurring for many years. It may also be due to nervous shock, sudden fright, intense anger, or any marked emotion. It is also not at all uncommon after bronchitis. In children this is specially so, and I may incidentally mention that sometimes in children the diagnosis between an attack of bronchitis and one of asthma is not always easy. But it is important to differentiate them because the treatment of the two diseases is so different.

### Ætiology.

One very important predisposing cause which I should mention is an abnormal condition of the nose or naso-pharynx. Some observers have been so impressed with the frequency with which these conditions have been found associated with asthma that they regard them as one of the main causes of asthma. There is a case in the Mount Vernon Hospital at the present time illustrating this condition rather well: that of a woman 44 years of age, who has had asthma for seven years, and who was found to have several nasal polypi. The first treatment was to give some drugs, which had a good effect, and then puncture of the antrum was done. No pus exuded, and yet the attacks were improved. A few days later nasal polypi were removed, and the attack then got less severe and less frequent.

The determining causes of asthma are so numerous that it is not practicable to go through them all with you; in fact, the idiosyncrasies of asthmatics are so various that you never can say what will be the most likely thing in any particular case to start an attack. One person will get an attack directly he moves from one locality to another. A woman may get an attack if there is a cat within range; some get an attack as soon as a horse comes anywhere near; others when they are in the presence of irritating fumes. Fog may bring on an attack. So you must find out from the patient what has acted as a determinant in his or her case.

The exact pathological cause is as yet undetermined. It is a disease which we speak of as functional, and which we shall continue to regard so until we find out something more definite. But it seems to be fairly well established that the condition is caused by a spasmodic contraction of the muscular coat of the bronchial tubes, and there is consequently a partial closure of the tubes. It is said this contraction may completely occlude the smaller tubes, so that there is great difficulty in drawing the air into the alveoli, and when it does get in it is difficult to get it out. Thus the alveoli get more and more distended with air, and the person has an excessive fulness of the alveoli. The expiratory act becomes considerably prolonged, so that a certain amount of air is got rid of, not because of the force with which it is sent out, but because time is given for it to trickle out, as it were. The reason for this spasm is supposed to be irritation of the ends of the vagi. There are so many things which may stimulate the terminations of the vagus nerve, which is distributed to various organs, that we can understand how varied are the causes which may start an attack.

It has been said that instead of there being a contraction of the muscular coats of the bronchi, there is a tumefaction of the lining membrane of the bronchial tubes, that they get congested and swollen, and that the blocking of these tubes is due to the swelling of the mucous membrane, rather than to the contraction of the muscular coat. Certainly there is tumefaction of the lining mucous membrane of the bronchial tubes during the attack of asthma; but probably this is the result of the asthmatic condition and not the cause of it. It has also been said that spasm of the diaphragm is the reason for the asthmatic seizure. But the evidence given in favour of that is by no means conclusive, and I think we may disregard it. The most commonly accepted cause is the spasmodic contraction of the muscular coat of the bronchial tubes: certainly the condition found during an attack corresponds with this assumption. It is important to bear in mind that view of the cause, because the treatment is very largely determined by it.

### Symptoms.

With regard to the symptoms of the complaint, an acute attack of asthma when once seen can hardly be forgotten. Sometimes the dyspnœa comes on suddenly, but at other times gradually; there may be something like the aura of epilepsy warning the patient that an attack is expected. Often an attack will come on in the middle of the night, and if there is only one attack in 24 hours it is more likely to be then. The dyspnœa quickly becomes more urgent, the patient is unable to lie down, so he sits up in bed, very likely leaning forward. Often he will rest his arms on some high object, such as a chest of drawers, and fix his upper limb so that the auxiliary muscles of respiration can be brought into play. The colour soon changes, the person first being pale and dusky, and then getting more and more ashy from the difficulty of getting the breath in, and therefore the want of aeration. A cold perspiration breaks out all over the body, the nostril is dilated, and the patient is unable to speak, but is fighting for his breath and as if he were getting the worst of it. This may go on for a couple of hours before relief comes. It exhausts the patient, but practically never are there fatal results. This is strange, seeing what a severe condition it is.

Now, if you come to examine that person in the ordinary way of physical examination—and, as a rule, it is unnecessary and tiresome to do so in a patient who is so suffering—you will find that the chest moves very little. There is hardly any movement of respiration, and the percussion note is hyper-resonant, the result of the over-distension of the lungs with air. With the stethoscope you scarcely hear any breathing sounds at all. What you will hear, probably, is only a very much prolonged and somewhat wheezy or sibilant expiratory sound. Practically the respiration sounds are absent. There will be some cough and some expectoration, and the latter is intensely viscid. If you examine this expectoration you find there are small lumps in it, which, when examined more carefully, may be unravelled into what are called spirals; a little central stem of very hardened mucus with, wound round it, a cylindrical shred of mucus, making Curschmann's spirals. You may also find a number of crystals. These masses of altered mucus are almost characteristic of the asthmatic attack, though you occasionally find them in some other conditions. As the attack is passing off the character of the expectoration alters, and you now find small pellets, looking like tapioca, coming up.

### Course.

The attack generally lasts about a couple of hours, but may last longer, and when it is over the person may go to sleep quickly, tired out and exhausted, and may wake up practically none the worse for it. Repeated attacks naturally lead to some alteration of the lungs, the chief of which is emphysema. There may be frequent recurrences of the attacks. A patient may have an attack for two or three nights, and then may go weeks, months, or possibly a year or two without a recurrence. Or you may come across people who have three or four attacks during the day, and they may be repeated every day for weeks. I had a case in hospital which I will mention to you now, that of a woman aged 43, who came in November last year. Since the beginning of that year she had not gone a single day without an asthmatic attack, usually one in the afternoon and one or two at night. They were extremely severe attacks. I saw a good number of the attacks, and she seemed no sooner to get out of one attack than she started another. She had, on the average, two or three in the 24 hours day after day for months. I shall mention that case again shortly in regard to treatment. One noticeable thing about the case is that the woman's family showed a very strong predisposition to tuberculosis. Her father died of phthisis, and her mother and one brother also. She herself had no signs of tuberculosis in the lungs, but she had had an attack of pleurisy at the beginning of that year. It was to that attack of pleurisy in January 1908 that she attributed her asthmatic attack. It is very uncommon for people who have tubercle of the lung to get asthma, and it is also uncommon for people subject to asthma to get tubercle of the lung. But I saw in consultation last year a tuberculous individual who had an asthmatic attack; he had early tuberculosis, and after being away for treatment he came back to London just in the middle of a severe fog. The result was that he had a sharp attack of asthma, which was typical in every respect, and was obviously caused by the irritation of the black fog.

### Diagnosis.

There is generally no difficulty in diagnosing an attack of true spasmodic asthma; those who have seen an attack will not have much difficulty in recognising the ordinary case. But there are one or two conditions which cause difficulties. I mentioned just now that in children bronchitis and asthma go together very much, and that in them it is sometimes difficult to distinguish between the two. In adults the chief difficulties may be in distinguishing between true asthma and these dyspnœic attacks which come in heart disease or in renal cases, or in distinguishing between true asthma and the effects of pressure by mediastinal tumour or aneurysm of the arch of the aorta, or from bronchial glands. I had a case only a few days ago which seemed to be one of asthma, and was sent to me rather with the idea that it was; but the conditions seemed to be an enlargement of bronchial glands which were pressing on the bronchi or vagus, and so setting up vagus attacks, which were considered to be asthmatic. Remember that pressure from bronchial glands in children may cause a spasmodic cough which so closely resembles whooping cough that it is often taken for it. The same pressure of glands may cause paroxysmal dyspnœa, instead of paroxysmal cough.

### Treatment.

Now we come to the more important question of treatment, because that is the main object of our study of disease. In talking of treatment we have to consider two points of view: First, the treatment of the attack, and then the treatment of the individual with the object of preventing further attacks. In the treatment of the attack we may aim at one of two things; we may aim at paralysing the terminations of the vagi, stimulation of which we supposed to be the starting-point of the condition; or we may use antispasmodics which do not directly act on the vagus terminations. Practically speaking, the drugs which we use may be divided into sedatives, depressants, and antispasmodics, though I grant that is not a very scientific classification. Those which act on the ends of the vagus include stramonium, conium, hyoscyamus, lobelia, atropine. Then we have chloroform, ether, bromide of potassium: morphine also is a sedative which is very valuable. Sometimes instead of using hypodermic injections of morphine, heroin is given with good results. Among the depressants there are ipecacuanha, pilocarpine, and tobacco. Most asthmatics will, if they get the opportunity, inhale some fumes during an attack, as for instance those from the burning of nitre paper or some similar preparation. Or they will smoke stramonium cigarettes, and even tobacco smoking tends to check an attack. In my own experience, when a person has a severe attack it is difficult to get him to smoke anything at all; it is rather more than he can manage. Of the antispasmodics, amyl nitrite is the one most chiefly employed, and it is certainly frequently of the greatest value. There are two more drugs which are not so commonly employed, but which I have found extremely useful. One is adrenalin, and the other paraldehyde. They seem to act as charms in some cases, and to check the attacks more surely and comfortably than anything else. When you have to deal with an asthmatic who has had the condition some years you find that he probably knows best what will check the attacks; he has tried so many remedies that at last he has come to the conclusion that one particular thing suits him best; and when such a patient gives you his experience you should not disregard it, as he probably knows by experience what you would have to find out by experiment. If you get a case for which many things have been tried I advise you to give a hypodermic injection of adrenalin chloride, injecting 5 minims of the 1 in 1,000 solution. That may be repeated if the attack does not stop. Paraldehyde may be given in 30 to 60 minim doses, and it sometimes stops an attack in a marvellous way.

In the case of the woman I have mentioned, who had these attacks day after day, a mixture of iodide of potassium 5 grs., ethereal tincture of lobelia 10 minims, tincture of stramonium 10 minims, chloroform water to 1 oz., given three times a day seemed to do a little towards diminishing the number of the attacks. And during the attack inhaling spirits of chloroform with compound tincture of benzoin seemed to give much relief, but did not check the attack. We then tried morphine injections, and they relieved the dyspnœa, but produced vomiting. We then tried the hypodermic injection of adrenalin chloride and found quick relief to the acute attack. In talking about the symptoms I might have mentioned that the pulse rate is considerably increased, and in this case it was from 100 to 124, and respirations 28 to 30, and sometimes as much as 38 per minute. A very good thing to give at the commencement of the attack is either a cup of strong coffee—even a cup of strong tea is useful—or you may give 10 grs. of nitrate of caffein instead of the cup of coffee. I mentioned chloroform inhalations, and they are frequently employed; but I warn you about chloroform inhalations for checking these attacks, as I have seen some rather alarming results from this procedure. Once or twice I have come across cases in which there seemed to be very serious risk from inhaling chloroform.

After a depressing illness a patient may be suffering from an acute attack of asthma, and one may be sent for because the patient is suffering from dyspnœa, which may have been going on for hours, and the patient seems likely to succumb from the difficulty in breathing; and in those cases I have found that the drugs which depress the circulation or arterial tension seem to be the most useful. But often the medical man in charge of the case, who, of course, has the responsibility when we have left, will be anxious about the effect of depressants in such a case, and it has often been difficult to convince the practitioner that he may give and push such depressants and not have any fear of the result. But where there is this fear it is not a bad thing to get a cylinder of oxygen, so that you may let the person inhale oxygen from time to time.

### After-treatment.

In between the paroxysms you will naturally try to safeguard the individual from the known determining cause. In young people frequently an attack may be brought on by a meal which has been either two large or badly selected. In such a case you will, of course, warn him accordingly. Diet is, in that sense, important between the paroxysms, but I do not think it need concern us otherwise.

The question of locality is one of some importance, because some people will have an attack in one place and be perfectly free in another; but your difficulty in selecting a locality is that it is impossible to say what particular kind of climate will suit your asthmatic. The patient must discover for himself which sorts of climate do not suit him. One has seen cases in which a short distance makes all the difference between having an attack and being free from them. When I was at University College Hospital a man came who was subject to asthma, and who, if he had an attack, jumped on to a bus and went up Hampstead Road, and when he reached the Cobden statue the attack ceased. The daughter of a medical man, friend of mine, in West Hampstead was subject to asthma, and when she had an attack her father took her on the underground railway—in the old days before it was well ventilated—and when they were in the part between Baker Street and King's Cross the attack ceased. Some asthmatics seemed to revel in the murky atmosphere of large towns, and in this case it was the worst atmosphere which could be found. One has heard of people having attacks of asthma when living on one side of the street, and being free when on the other side. It is difficult to know where any particular patient should live; but his own experience tells him.

The Question of Drugs.

The two drugs which are chiefly relied upon to diminish the tendency to these attacks are arsenic and iodide of potassium. A course of arsenic seems to do a great deal for many cases, and so does iodide of potassium, though with the latter the liability to attack does not seem to be diminished, though the frequency does. If the drug is given up the attacks seem to recur with the old frequency. I have used much larger doses than the usual 5 grs., and do not hesitate to do so if the smaller ones have no effect. I have given as much as 60 grs. three times a day for some weeks, and while it was taken the attacks were completely kept off. But I do not think there is any occasion to use such heroic doses. Many people get dyspnœa, cough, and various discomforts at the age when their arteries become somewhat degenerated, who may be made comfortable by small doses of iodide which keep down the blood-pressure, but large doses make them miserable. In such cases it is well to diminish the dose to 1 gr. or 2 grs.

Cases of gouty asthma, or symptomatic asthma are generally relieved by keeping the patient on small doses of iodide, for they seem to be determined by high blood-pressure. But for true spasmodic asthma I think iodide should be used only for the moment, and not be relied upon to check the attacks: arsenic is much more likely to have permanent effects. In the case of children there is reason to hope that as they get older they may get rid of the attacks, especially about the time of puberty. In adults the chance of getting rid of the attacks is smaller. In older people the attacks may be kept under by iodide, but after middle-age the disease tends to go on, whatever drugs we may use.

